# Supplementary material for: Chronic oral cannabidiol delays seizure onset and reduces seizure burden in a mouse model of CLN2 disease
Source: PLoS One. 2026 Jul 20;21(7):e0337880. doi: 10.1371/journal.pone.0337880 (PMC13384314; doi:10.1371/journal.pone.0337880)
Supplement: Attachment — Submitted filename: Response to Reviewers.docx [file pone.0337880.s003.docx]

Editors and Reviewers,

As instructed, this letter lists each point raised by an editor or reviewer to which we have responded, and includes the location, method, and/or explanation for the response.

Points raised by the Editors:

1. I have ensured that the manuscript meets PLOS ONE’s style requirements, including those for file naming. I followed the style templates provided.
2. Here is our amended Funding Statement, as requested:

*JTD and JDC received generous gifts from the Lehrman Family Fund to conduct this research. Special thanks to Stephen and Sandra Lehrman for their support. JDC also received funding from the Batten Disease Support and Research Association to support his research. MW received funding from the Washington University in St. Louis IDDRC – NIH P50 HD103525. There was no additional external funding received for this study.*

3. We have amended the Acknowledgements sections as requested, and it no longer contains any funding information that appears in our Funding Statement. Our new Funding statement can be seen in the point above.
4. We will happily comply with the data compliance requirements as described, however the text provided in the review is unclear. We have already indicated that we will make our data available on acceptance, and were then recommended that all authors decide on a data sharing plan before acceptance. We have already agreed on the plan, and will execute upon acceptance. Our data will be made freely accessible upon acceptance, and we will host it on an accessible database supported by our University. A link will be provided. I do not believe we need to amend our data availability statement; we are still committed to adhering to your open data policy.
5. No Number 5 was included in the Editor’s list.
6. We have removed the phrase “data not shown” in the one instance where we originally used it, line 120. We have added reference to a Supplementary Table showing the data collected and added this table to the Supporting Information section at the end of our revised manuscript (line 551). We will submit the Supplementary table with our resubmission.
7. We have added an “Ethics statement” section to our Methods section and included our ethics statement and the full name of the IRB who approved our study. (Line 95)

Points raised by Reviewer 1:

1. We have changed the title of the manuscript as suggested. (line 3)
2. We have changed the wording of “nearly significant” in the abstract to better represent the statistical difference referenced. (line 34)
3. We have added a statement about our primary aim and secondary aims, to clarify the question. (line 90)
4. In the Introduction, we have added a statement about the translational justification for the selected dose, the approximate half-life of CBD in rodents and humans, and references to support the statement and our dose. (line 86) This was also added to the CBD administration section in the Methods. (line 134). We believe it important to note that the original Discussion also includes rationale for the dosage (not like 518), and that this remains.
5. In each Methods subsection, it was noted that animals were assigned by a blinded third party and that measurements were taken by someone blinded to treatment status.
6. Regarding CBD administration and intake monitoring, we have included data from a pilot study that we ran to confirm that CLN2 mice would eat a gelatin cube with drug in it. This was performed over 9 consecutive days using 5 CLN2 mice, and we measured the amount of cube consumed. This data is now included as a Supplemental Table. (line 131).
7. We have clarified in our Ethics Statement (line 105) that the sodium pentobarbitol product used was Fatal-Plus, when it was used, and also explained the CO2 induction was used to euthanize EEG mice when they met the humane endpoint.
8. In the Statistical Analysis sections, we have defined the primary and secondary endpoints as requested. (line 252)
9. We believe that the request for rationale of parametric versus nonparametric tests are met: on line 250 we detail that the t-test was used when assumptions of normality were met, and that a nonparametric test was employed with the data was distributed non-normally.
10. We greatly appreciate the point brought up regarding pseudoreplication. It was never our intention to manipulate or misrepresent our objective findings in a way that would obscure the statistics or portray them inappropriately. Regardless of our intention, we acknowledge the issue and thank the reviewer for their insight. To resolve this issue we have taken multiple steps. First, we removed the original panel Fig. 3c which showed the duration of each individual seizure event; this was the graph that included multiple seizure events from the same animal. This figure and data has been omitted. We have kept our animal-level analysis in original figures 3d and 3e, and they remain as the reported findings. Second, beginning on line 348 in the Discussion we have included a paragraph describing the nature of the current study and the rationale for displaying the data as we do (now with 3c omitted). This explanation clarifies that given the paucity of knowledge of CBD’s mechanism and the unknown etiology of seizures in the CLN2 disease, we sought not to administer a dose-response study or to even explain what an exact dose does in a mouse of a specific weight. We have clarified that we sought to learn the effects of a moderately-high universal dose on the broad seizure phenotype in the CLN2 mouse, and tried to convey that there is an exploratory nature to the current project.
11. As requested, individual data points from mass spec findings are reported (line 262), and timing relative to last ingestion is detailed (line 264).
12. We have added detail to how thresholding was defined, number of sections per animal, and the blinded nature of analysis in the Neuroinflammatory markers section (line 175 and beyond).
13. We have added details to the Cytokine/Chemokine Methods to be clear that we performed these analyses in an exploratory manner. We take to heart the reviewer’s concern about false-positives and appropriate correction, and we would apply such a correctly if we had substantial significant findings in this arena or brought specific hypotheses about particular analytes. As we were using this mini-panel as a cursory exploration of immune response, we have tried to explicate our standing. (line 208).
14. We have removed the Lifespan comparison that employed mean days, and what remains of the lifespan results is the Kaplan-Meier survival analysis.
15. We have explicitly acknowledged a number of limitations of the study, as requested, by adding a paragraph in the discussion (line 502).
16. We have re-worded the Conclusion section at the suggestion provided, which we appreciate. (line 527)
17. Sample sizes have been added to all figures and figure numbering has been checked and corrected for consistency.
18. The duplicate in the references has been omitted, and all reference numbers have been updated.

Points raised by Reviewer 2:

1. We appreciate and understand the insight offered by the reviewer with regard to recent preclinical studies reporting antiseizure effects of CBD at significantly lower doses. As requested, we have consulted the papers suggested and integrated them into a brief discussion of dosing and disparate results between our study here and some previous ones (beginning line 439). We also note that the dosing and pharmacokinetics are discussed in lines 530-535 of the Discussion.
2. We have addressed the variability introduced by our voluntary oral administration strategy, as requested. Such discussion is located in sections beginning on 132, line 354, and line 476.
3. We have added notes about lack of pathological benefit in the context of delayed seizure onset, and that future studies would benefit from taking such measures at multiple timepoints to more precisely probe the effects of CBD on both pathology and seizure phenotype (line 435). We have also added suggestion that future studies explore multiple daily treatments, as requested. (line 554)
4. In the Animals section (line 109) we have noted sex distribution in each cohort of mice. We have also remarked on sex-dependent effects, and that we were underpowered to perform such analysis but that future studies would benefit from such an endeavor (line 485).
5. We included a detailed figure caption to make clear the symbols and endpoints in Figure 3, which has also now been cut from 7 panels to 5. We also increased the size of panel b in Figure 3, and included text describing it in detail within the Results section (line 380).
6. We have defined terminology for the mouse model (line 73) to make clear that all references pertain to the same mouse.

Thank you for your work and consideration.

Joshua T. Dearborn
