# Supplementary material for: Chronic oral cannabidiol delays seizure onset and reduces seizure burden in a mouse model of CLN2 disease
Source: PLoS One. 2026 Jul 20;21(7):e0337880. doi: 10.1371/journal.pone.0337880 (PMC13384314; doi:10.1371/journal.pone.0337880)
Supplement: Peer review history

# Peer review: Decision Letter 0

**DOI:** 10.1371/journal.pone.0337880.r001

**Authors:** Giuseppe Biagini

28 Jan 2026

PONE-D-25-61424

Chronic oral cannabidiol delays or prevents seizures in a mouse model of CLN2 disease

PLOS One

Dear Dr. Dearborn,

Thank you for submitting your manuscript to PLOS ONE. After careful consideration, we feel that it has merit but does not fully meet PLOS ONE’s publication criteria as it currently stands. Therefore, we invite you to submit a revised version of the manuscript that addresses the points raised during the review process.

Please submit your revised manuscript by Mar 14 2026 11:59PM. If you will need more time than this to complete your revisions, please reply to this message or contact the journal office at plosone@plos.org. When you're ready to submit your revision, log on to https://www.editorialmanager.com/pone/ and select the 'Submissions Needing Revision' folder to locate your manuscript file.

Please include the following items when submitting your revised manuscript:

- A letter that responds to each point raised by the academic editor and reviewer(s). You should upload this letter as a separate file labeled 'Response to Reviewers'.
- A marked-up copy of your manuscript that highlights changes made to the original version. You should upload this as a separate file labeled 'Revised Manuscript with Track Changes'.
- An unmarked version of your revised paper without tracked changes. You should upload this as a separate file labeled 'Manuscript'.

If you would like to make changes to your financial disclosure, please include your updated statement in your cover letter. Guidelines for resubmitting your figure files are available below the reviewer comments at the end of this letter.

If applicable, we recommend that you deposit your laboratory protocols in protocols.io to enhance the reproducibility of your results. Protocols.io assigns your protocol its own identifier (DOI) so that it can be cited independently in the future. For instructions see: https://journals.plos.org/plosone/s/submission-guidelines#loc-laboratory-protocols. Additionally, PLOS ONE offers an option for publishing peer-reviewed Lab Protocol articles, which describe protocols hosted on protocols.io. Read more information on sharing protocols at https://plos.org/protocols?utm_medium=editorial-email&utm_source=authorletters&utm_campaign=protocols.

We look forward to receiving your revised manuscript.

Kind regards,

Giuseppe Biagini, MD

Academic Editor

PLOS One

Journal Requirements:

When submitting your revision, we need you to address these additional requirements.

1. Please ensure that your manuscript meets PLOS ONE's style requirements, including those for file naming. The PLOS ONE style templates can be found at

https://journals.plos.org/plosone/s/file?id=wjVg/PLOSOne_formatting_sample_main_body.pdf and https://journals.plos.org/plosone/s/file?id=ba62/PLOSOne_formatting_sample_title_authors_affiliations.pdf

2. Thank you for stating in your Funding Statement:

[JTD and JDC received generous gifts from the Lehrman Family Fund to conduct this research. Special thanks to Stephen and Sandra Lehrman for their support.

JDC also received funding from the Batten Disease Support and Research Association to support this research.

MW received funding from the Washington University in St. Louis IDDRC - NIH P50 HD103525].

Please provide an amended statement that declares *all* the funding or sources of support (whether external or internal to your organization) received during this study, as detailed online in our guide for authors at http://journals.plos.org/plosone/s/submit-now.  Please also include the statement “There was no additional external funding received for this study.” in your updated Funding Statement.

Please include your amended Funding Statement within your cover letter. We will change the online submission form on your behalf.

3. Thank you for stating the following in the Acknowledgments Section of your manuscript:

[The authors give a special thanks to Stephen and Sandra Lehrman and the Lehrman Family Fund for providing financial support to complete this work. Thanks also to the Batten Disease Support and Research Association for research support, and to Dr. Russell B. Williams at the Proteomics and Mass Spectrometry Facility of the Donald Danforth Plant Science Center who performed LC-MS/MS analysis on instruments purchased with DDPSC funds.]

We note that you have provided funding information that is currently declared in your Funding Statement. However, funding information should not appear in the Acknowledgments section or other areas of your manuscript. We will only publish funding information present in the Funding Statement section of the online submission form.

Please remove any funding-related text from the manuscript and let us know how you would like to update your Funding Statement. Currently, your Funding Statement reads as follows:

[JTD and JDC received generous gifts from the Lehrman Family Fund to conduct this research. Special thanks to Stephen and Sandra Lehrman for their support.

JDC also received funding from the Batten Disease Support and Research Association to support this research.

MW received funding from the Washington University in St. Louis IDDRC - NIH P50 HD103525]

Please include your amended statements within your cover letter; we will change the online submission form on your behalf.

4. When completing the data availability statement of the submission form, you indicated that you will make your data available on acceptance. We strongly recommend all authors decide on a data sharing plan before acceptance, as the process can be lengthy and hold up publication timelines. Please note that, though access restrictions are acceptable now, your entire data will need to be made freely accessible if your manuscript is accepted for publication. This policy applies to all data except where public deposition would breach compliance with the protocol approved by your research ethics board. If you are unable to adhere to our open data policy, please kindly revise your statement to explain your reasoning and we will seek the editor's input on an exemption. Please be assured that, once you have provided your new statement, the assessment of your exemption will not hold up the peer review process.

6. We note that you have included the phrase “data not shown” in your manuscript. Unfortunately, this does not meet our data sharing requirements. PLOS does not permit references to inaccessible data. We require that authors provide all relevant data within the paper, Supporting Information files, or in an acceptable, public repository. Please add a citation to support this phrase or upload the data that corresponds with these findings to a stable repository (such as Figshare or Dryad) and provide and URLs, DOIs, or accession numbers that may be used to access these data. Or, if the data are not a core part of the research being presented in your study, we ask that you remove the phrase that refers to these data.

7. Please include your full ethics statement in the ‘Methods’ section of your manuscript file. In your statement, please include the full name of the IRB or ethics committee who approved or waived your study, as well as whether or not you obtained informed written or verbal consent. If consent was waived for your study, please include this information in your statement as well.

If the reviewer comments include a recommendation to cite specific previously published works, please review and evaluate these publications to determine whether they are relevant and should be cited. There is no requirement to cite these works unless the editor has indicated otherwise.

[Note: HTML markup is below. Please do not edit.]

Reviewers' comments:

Reviewer's Responses to Questions

**Comments to the Author**

1. Is the manuscript technically sound, and do the data support the conclusions?

The manuscript must describe a technically sound piece of scientific research with data that supports the conclusions. Experiments must have been conducted rigorously, with appropriate controls, replication, and sample sizes. The conclusions must be drawn appropriately based on the data presented.

Reviewer #1: Yes

Reviewer #2: Partly

**********

2. Has the statistical analysis been performed appropriately and rigorously?

Reviewer #1: N/A

Reviewer #2: Yes

**********

3. Have the authors made all data underlying the findings in their manuscript fully available?

The PLOS Data policy requires authors to make all data underlying the findings described in their manuscript fully available without restriction, with rare exception (please refer to the Data Availability Statement in the manuscript PDF file). The data should be provided as part of the manuscript or its supporting information, or deposited to a public repository. For example, in addition to summary statistics, the data points behind means, medians and variance measures should be available. If there are restrictions on publicly sharing data—e.g. participant privacy or use of data from a third party—those must be specified.

Reviewer #1: Yes

Reviewer #2: Yes

**********

4. Is the manuscript presented in an intelligible fashion and written in standard English?

PLOS ONE does not copyedit accepted manuscripts, so the language in submitted articles must be clear, correct, and unambiguous. Any typographical or grammatical errors should be corrected at revision, so please note any specific errors here.

Reviewer #1: Yes

Reviewer #2: Yes

**********

5. Review Comments to the Author

Please use the space provided to explain your answers to the questions above. You may also include additional comments for the author, including concerns about dual publication, research ethics, or publication ethics. (Please upload your review as an attachment if it exceeds 20,000 characters)

Reviewer #1: The manuscript addresses an important and clinically relevant question, namely whether chronic oral cannabidiol (CBD) can delay seizure onset and reduce seizure burden in a mouse model of CLN2 disease (Cln2R207X). The study has notable strengths, including the use of continuous video-EEG monitoring and objective outcome measures such as time to first seizure and survival. The observation that CBD delays seizure onset and that a subset of treated animals remains seizure-free is potentially meaningful.

However, in its current form, the manuscript presents several methodological and analytical issues that limit the robustness of the conclusions. In particular, concerns related to statistical treatment (especially pseudoreplication), multiple-comparison control, incomplete reporting of randomization and blinding procedures, inconsistencies in ethical descriptions, and incomplete characterization of dosing and intake must be addressed before the work can be considered for publication. For these reasons, I recommend major revision.

Section-by-section comments

Title and Abstract

The title is generally appropriate, but the wording “delays or prevents seizures” may overstate the findings. Since seizure prevention was observed only in a subset of animals, a more cautious formulation (e.g., “delays seizure onset and reduces seizure burden”) would be more accurate.

In the abstract, the use of expressions such as “nearly significant” (e.g., regarding survival) should be avoided. Results with p values above the significance threshold should be described as not statistically significant, ideally accompanied by effect size estimates or confidence intervals. In addition, it should be clearly stated that the reported dose of 300 mg/kg represents a target or estimated dose, based on voluntary consumption, rather than a precisely administered amount.

Introduction

The introduction provides a solid background on CLN2 disease and the rationale for evaluating CBD. However, the main hypothesis should be stated more explicitly. It is currently unclear whether the study primarily aims to test an anticonvulsant effect, an anti-inflammatory mechanism, or both.

The translational justification for the selected dose also requires clarification. The equivalence between the mouse dose and an approximate human dose should be supported with appropriate references and accompanied by a discussion of the limitations inherent to interspecies scaling and oral bioavailability differences.

Methods

This section requires substantial clarification to meet standards of reproducibility and rigor.

Randomization and blinding.

The manuscript should explicitly state whether animals were randomly assigned to treatment groups and describe the randomization procedure used. It should also clarify whether investigators performing EEG analysis, histological quantification, and cytokine measurements were blinded to group allocation.

CBD administration and intake monitoring.

Because CBD was administered via a gelatin-based edible with reported consumption of approximately 60–75%, it is essential to provide the actual intake data. These data should ideally be included as supplementary material, showing variability by animal and by sex, and explaining how the effective dose was calculated and adjusted over time. Without this information, it is difficult to interpret exposure consistency.

Ethical procedures and euthanasia.

There are inconsistencies in the manuscript regarding euthanasia methods (references to pentobarbital, Fatal-Plus, and CO₂). These should be reconciled, clearly indicating which methods were used for which experimental groups and confirming that all procedures were approved under the same institutional protocol.

Statistical analysis.

The statistical section requires substantial revision. The primary endpoint should be clearly defined (e.g., time to first seizure). Secondary and exploratory outcomes should be explicitly labeled as such.

Effect sizes and confidence intervals should be reported where applicable (e.g., hazard ratios for survival analyses). The rationale for choosing parametric versus nonparametric tests should be clarified.

Most importantly, the manuscript must address the issue of pseudoreplication. Analyses of seizure duration appear to treat individual seizures as independent observations, even though multiple seizures originate from the same animal. This violates the assumption of independence. These data should be reanalyzed using either:

animal-level summaries (e.g., median seizure duration per animal), or

mixed-effects or clustered models with animal as a random effect.

Without such correction, conclusions regarding seizure duration are not statistically valid.

Results

Brain CBD concentrations.

Measurement of brain CBD levels by LC–MS/MS is a strength of the study. However, additional information is needed regarding the timing of tissue collection relative to last ingestion, inter-animal variability, and whether sampling was standardized. Displaying individual data points would improve transparency.

Neuroinflammatory markers (GFAP and CD68).

Negative findings are acceptable, but methodological details require clarification, including how thresholding was defined, how many sections per animal were analyzed, and whether quantification was performed blinded. Reporting effect sizes and confidence intervals, even in the absence of significance, would strengthen interpretation.

Cytokine and chemokine panel.

This section raises a major concern due to multiple testing. With 12 analytes analyzed, the risk of false-positive findings is substantial. Appropriate correction for multiple comparisons (e.g., false discovery rate) should be applied. Alternatively, the authors should clearly define these analyses as exploratory. Claims regarding IL-1β, IL-6, or GROα should be interpreted cautiously unless corrected analyses support them.

Seizure outcomes.

The delay in time to first seizure is a key strength of the study and should be presented with full survival analysis, including numbers at risk and, if possible, hazard ratios with confidence intervals.

The analysis of seizure duration requires reanalysis, as noted above, to avoid pseudoreplication. The current degrees of freedom strongly suggest that individual seizures were treated as independent observations, which is inappropriate.

Survival analysis.

The manuscript inconsistently reports survival using both a comparison of mean days and a log-rank test. The survival analysis should be standardized, prioritizing Kaplan–Meier curves and Cox regression where applicable. Statements implying “near significance” should be avoided.

Discussion

The discussion is generally balanced but should further temper mechanistic interpretations. While the absence of changes in GFAP, CD68, or cytokines is acknowledged, conclusions should avoid implying mechanistic anti-inflammatory effects. These should instead be framed as hypotheses for future investigation.

Limitations should be more explicitly acknowledged, including:

variability in actual CBD intake,

lack of dose–response evaluation,

small sample sizes for pharmacokinetic measurements,

absence of sex-stratified analyses,

limited power for survival outcomes.

Conclusions

The conclusions should be revised to reflect the data more conservatively. A suggested framing would be that CBD administration was associated with delayed seizure onset and that a subset of animals remained seizure-free during the observation period, while no clear modulation of glial or inflammatory markers was detected under the conditions tested.

Figures, consistency, and references

All figures should clearly report sample sizes. Kaplan–Meier plots should include numbers at risk. Figure numbering should be checked for consistency, as discrepancies appear between the text and figure labels.

The reference list should be reviewed for duplication and formatting inconsistencies (notably repeated citations related to gene therapy studies).

Reviewer #2: This manuscript by Dearborn et al. examines the effects of chronic oral CBD (300 mg/kg) in mouse model of CLN2 disease. The authors report that long-term CBD treatment significantly delays or prevents seizure onset and leads to a near-significant extension of lifespan, despite no improvement in classical neuroinflammatory or neuroimmune markers. The study addresses a clinically relevant question, and provides evidence for an anti-seizure effect of CBD. However, some aspects of the experimental design, data analysis, and interpretation require clarification to strengthen the conclusions.

• The study employs a single relatively high dose of CBD (300 mg/kg) without a dose–response analysis, and this approach limits interpretation of both efficacy and therapeutic window. Notably, recent preclinical studies have reported antiseizure effects of CBD at substantially lower doses (120 mg/kg), including dose-dependent effects (e.g., 12 vs 120 mg/kg) and subthreshold dosing strategies (once vs twice a day). The authors are encouraged to discuss these studies in more detail, comparing their dosing strategy with existing literature and clarifying whether the chosen dose reflects disease severity, pharmacokinetic considerations, or other experimental constraints. Please see:

Costa, AM. et al, Antiseizure Effects of Fully Characterized Non-Psychoactive Cannabis sativa L. Extracts in the Repeated 6-Hz Corneal Stimulation Test. Pharmaceuticals 2021, 14, 1259. https://doi.org/10.3390/ph14121259

Costa, AM. et al, Antiseizure Effects of Cannabidiol Leading to Increased Peroxisome Proliferator-Activated Receptor Gamma Levels in the Hippocampal CA3 Subfield of Epileptic Rats. Pharmaceuticals 2022,15, 495. https://doi.org/10.3390/ph15050495

Lucchi, C. et al, Subthreshold Cannabidiol Potentiates Levetiracetam in the Kainic Acid Model of Temporal Lobe Epilepsy: A Pilot Study. Pharmaceuticals 2024, 17, 1187. https://doi.org/10.3390/ph17091187

• Voluntary oral delivery via gelatin cubes likely introduces variability in individual drug intake. The authors should clarify whether intake was monitored and discuss how this variability may affect seizure-related outcomes.

• The primary outcome (time to first seizure) shows a significant treatment effect, whereas seizure frequency and seizure duration display limited or only marginal effects. The authors should discuss possible reasons for this outcome. In particular, it should be consider whether the dosing regimen may have influenced seizure frequency outcomes. Notably, preclinical studies reporting reductions in seizure number have employed multiple daily administrations of CBD (e.g., twice-daily dosing at ~12 h intervals) to achieve more stable drug exposure and better mimic clinical treatment paradigms. Finally, the authors should clarify whether EEG analysis and seizure scoring were performed blinded to treatment condition. Please see Costa et al., 2022.

• Indicate sex distribution across groups and whether sex-dependent effects were assessed.

• Improve clarity of Figure 3 (especially panel b) regarding symbols and endpoints.

• Standardize terminology for the CLN2 mouse model throughout the manuscript.

**********

6. PLOS authors have the option to publish the peer review history of their article (what does this mean?). If published, this will include your full peer review and any attached files.

If you choose “no”, your identity will remain anonymous but your review may still be made public.

**Do you want your identity to be public for this peer review?**  For information about this choice, including consent withdrawal, please see our Privacy Policy.

Reviewer #1: No

Reviewer #2: No

**********

[NOTE: If reviewer comments were submitted as an attachment file, they will be attached to this email and accessible via the submission site. Please log into your account, locate the manuscript record, and check for the action link "View Attachments". If this link does not appear, there are no attachment files.]

To ensure your figures meet our technical requirements, please review our figure guidelines: https://journals.plos.org/plosone/s/figures

You may also use PLOS’s free figure tool, NAAS, to help you prepare publication quality figures: https://journals.plos.org/plosone/s/figures#loc-tools-for-figure-preparation.

NAAS will assess whether your figures meet our technical requirements by comparing each figure against our figure specifications.

# Author comment: Author response to Decision Letter 1

**DOI:** 10.1371/journal.pone.0337880.r002

19 Apr 2026

All reviewer and editor comments in the Decision Letter have been addressed and are detailed in the Response to Reviewers letter attached with the submission.

Attachment Submitted filename: Response to Reviewers.docx

# Peer review: Decision Letter 1

**DOI:** 10.1371/journal.pone.0337880.r003

**Authors:** Giuseppe Biagini

12 Jun 2026

Chronic oral cannabidiol delays seizure onset and reduces seizure burden in a mouse model of CLN2 disease

PONE-D-25-61424R1

Dear Dr. Dearborn,

We’re pleased to inform you that your manuscript has been judged scientifically suitable for publication and will be formally accepted for publication once it meets all outstanding technical requirements.

Within one week, you’ll receive an e-mail detailing the required amendments. When these have been addressed, you’ll receive a formal acceptance letter and your manuscript will be scheduled for publication.

An invoice will be generated when your article is formally accepted. Please note, if your institution has a publishing partnership with PLOS and your article meets the relevant criteria, all or part of your publication costs will be covered. Please make sure your user information is up-to-date by logging into Editorial Manager at Editorial Manager® and clicking the ‘Update My Information' link at the top of the page. For questions related to billing, please contact billing support.

If your institution or institutions have a press office, please notify them about your upcoming paper to help maximize its impact. If they’ll be preparing press materials, please inform our press team as soon as possible -- no later than 48 hours after receiving the formal acceptance. Your manuscript will remain under strict press embargo until 2 pm Eastern Time on the date of publication. For more information, please contact onepress@plos.org.

Kind regards,

Giuseppe Biagini, MD

Academic Editor

PLOS One

Additional Editor Comments (optional):

Reviewers' comments:

Reviewer's Responses to Questions

**Comments to the Author**

1. If the authors have adequately addressed your comments raised in a previous round of review and you feel that this manuscript is now acceptable for publication, you may indicate that here to bypass the “Comments to the Author” section, enter your conflict of interest statement in the “Confidential to Editor” section, and submit your "Accept" recommendation.

Reviewer #1: All comments have been addressed

**********

2. Is the manuscript technically sound, and do the data support the conclusions?

The manuscript must describe a technically sound piece of scientific research with data that supports the conclusions. Experiments must have been conducted rigorously, with appropriate controls, replication, and sample sizes. The conclusions must be drawn appropriately based on the data presented.

Reviewer #1: Yes

**********

3. Has the statistical analysis been performed appropriately and rigorously?

Reviewer #1: Yes

**********

4. Have the authors made all data underlying the findings in their manuscript fully available?

The PLOS Data policy requires authors to make all data underlying the findings described in their manuscript fully available without restriction, with rare exception (please refer to the Data Availability Statement in the manuscript PDF file). The data should be provided as part of the manuscript or its supporting information, or deposited to a public repository. For example, in addition to summary statistics, the data points behind means, medians and variance measures should be available. If there are restrictions on publicly sharing data—e.g. participant privacy or use of data from a third party—those must be specified.

Reviewer #1: Yes

**********

5. Is the manuscript presented in an intelligible fashion and written in standard English?

PLOS ONE does not copyedit accepted manuscripts, so the language in submitted articles must be clear, correct, and unambiguous. Any typographical or grammatical errors should be corrected at revision, so please note any specific errors here.

Reviewer #1: Yes

**********

6. Review Comments to the Author

Please use the space provided to explain your answers to the questions above. You may also include additional comments for the author, including concerns about dual publication, research ethics, or publication ethics. (Please upload your review as an attachment if it exceeds 20,000 characters)

Reviewer #1: (No Response)

**********

7. PLOS authors have the option to publish the peer review history of their article (what does this mean?). If published, this will include your full peer review and any attached files.

If you choose “no”, your identity will remain anonymous but your review may still be made public.

**Do you want your identity to be public for this peer review?**  For information about this choice, including consent withdrawal, please see our Privacy Policy.

Reviewer #1: No

**********

# Editor report: Acceptance letter

**DOI:** 10.1371/journal.pone.0337880.r004

**Authors:** Giuseppe Biagini

PONE-D-25-61424R1

PLOS One

Dear Dr. Dearborn,

I'm pleased to inform you that your manuscript has been deemed suitable for publication in PLOS One. Congratulations! Your manuscript is now being handed over to our production team.

At this stage, our production department will prepare your paper for publication. This includes ensuring the following:

* All references, tables, and figures are properly cited

* All relevant supporting information is included in the manuscript submission,

* There are no issues that prevent the paper from being properly typeset

You will receive further instructions from the production team, including instructions on how to review your proof when it is ready. Please keep in mind that we are working through a large volume of accepted articles, so please give us a few days to review your paper and let you know the next and final steps.

Lastly, if your institution or institutions have a press office, please let them know about your upcoming paper now to help maximize its impact. If they'll be preparing press materials, please inform our press team within the next 48 hours. Your manuscript will remain under strict press embargo until 2 pm Eastern Time on the date of publication. For more information, please contact onepress@plos.org.

You will receive an invoice from PLOS for your publication fee after your manuscript has reached the completed accept phase. If you receive an email requesting payment before acceptance or for any other service, this may be a phishing scheme. Learn how to identify phishing emails and protect your accounts at https://explore.plos.org/phishing.

If we can help with anything else, please email us at customercare@plos.org.

Thank you for submitting your work to PLOS ONE and supporting open access.

Kind regards,

PLOS ONE Editorial Office Staff

on behalf of

Dr. Giuseppe Biagini

Academic Editor

PLOS One
